# Effect of mannitol on diuresis and acid–base equilibrium in critically ill patients

**DOI:** 10.1186/s40635-025-00807-y

**Published:** 2025-09-16

**Authors:** Davide Chiumello, Clara Aiello, Tommaso Pozzi, Francesca Panina, Alessandra Muscas, Simone Mancusi, Silvia Coppola

**Affiliations:** 1 Department of Anesthesia and Intensive Care, ASST Santi Paolo E Carlo, San Paolo University Hospital, Milan, Italy; 2 Department of Health Sciences, University of Milan, Milan, Italy; 3 Coordinated Research Center On Respiratory Failure, University of Milan, Milan, Italy

**Keywords:** Mannitol, Acid–base disorders, Urine electrolytes, Strong ion difference

## Abstract

**Background:**

Mannitol is the most commonly used osmotic diuretic, but its effect on plasma and urine electrolytes and on acid–base equilibrium have not been well investigated. The aim of this study was to evaluate the short-term effects of mannitol on diuresis and plasma and urine acid–base equilibrium in a group of critically ill patients according to the Stewart approach.

**Results:**

Prospective observational study enrolling all consecutive sedated and mechanically ventilated patients requiring mannitol infusion for clinical purpose. Plasma and urine acid–base variables and electrolytes were measured before mannitol infusion and every 60 and 30 min, respectively, following the infusion of 1 g/kg of ideal body weight of mannitol. Forty-two patients were enrolled. Diuresis increased significantly 30 min after the mannitol infusion was completed and remained significantly higher as compared with T_0_. Plasma sodium and chloride concentrations and plasma SID significantly decreased after mannitol infusion ended; urine sodium and chloride concentration remained unchanged, while urine ammonium increased increasing urine SID.

**Conclusions:**

Since the end of the infusion, mannitol promoted a significant increase in diuresis, with a reduction in plasma electrolytes due to volume expansion, and a slight decrease in arterial pH due to dilutional acidosis. Kidney relative excretion pattern was unmodified during the study.

## Introduction

Mannitol is the most commonly used osmotic diuretic. Its clinical applications include increasing diuresis, reducing intracranial pressure, excreting toxins and preventing acute renal failure in patients with rhabdomyolysis or following the radiocontrast use [1–3].

Mannitol is a non-metabolizable six-carbon polyalcohol with a molecular weight of 182 g/mol. When administered intravenously, it is distributed primarily into the extracellular fluid compartment (both intravascular and extravascular), without crossing the blood–brain barrier [1, 2, 4]. Mannitol is freely filtered at the glomerular level, but is poorly reabsorbed at the tubular site. In the absence of renal failure, up to 90% of mannitol is excreted by the kidneys unmetabolized within a few hours [1, 5]. Following intravascular infusion, mannitol increases plasma osmolality, drawing water from the intracellular and interstitial spaces into the intravascular compartment [2, 6]. In the kidney, it generates an osmotic force in the tubular fluid that suppresses the reabsorption of fluid and solutes. This increases diuresis and the possible loss of electrolytes [2, 4, 7]. In neurosurgical patients, it was found that mannitol significantly reduced serum sodium and haemoglobin concentrations, while increasing osmolality 15 min after infusion ended. These changes persisted for up to 60 min [6, 8]. Furthermore, a significant correlation was found between mannitol dosage and changes in serum osmolality. Following infusion, mannitol initially promotes plasma volume expansion, followed by a diuretic phase that can cause electrolytes imbalances, such as hypo- or hyper-natremia, hypokalemia, hypocalcaemia and hypomagnesaemia [2]. Additionally, the possible volume depletion due to the osmotic diuresis can activate the renin–angiotensin-aldosterone system (RASS), leading to water and sodium retention [7].

Therefore, the ultimate effect of mannitol depends on the patient’s initial plasma volume, the extent of diuresis, the status of the kidneys and the level of activation of the RASS system. The potential changes in plasma and urine electrolyte composition and acid–base equilibrium following mannitol infusion have not yet been thoroughly investigated.

Clinicians commonly evaluate acid–base equilibrium using the Henderson-Hasselbalch equation, which considers carbon dioxide and actual bicarbonate concentration to be the only determinants of pH [9]. However, this approach does not consider bicarbonate independently of the respiratory component and fails to quantify other buffers [10].

To overcome these limitations the Stewart approach has been proposed [11]. This model assumes that plasma pH is determined by three independent variables: the strong ion difference (SID), which is the difference between the fully dissociated plasm strong cations (sodium, potassium, calcium, magnesium) and plasma strong anions (chloride and lactate); the total weak acid concentration (A_TOT_), which mainly consists of albumin and phosphate; and the partial pressure of carbon dioxide. According to this model, a full evaluation of acid–base status requires an arterial blood gas analysis as well as laboratory measurements of serum electrolytes and albumin and phosphate concentration. However, recent studies have shown that there is poor agreement between the Stewart approach and the Henderson-Hasselbalch approach in critically ill patients [12].

The present study aimed to evaluate the short-term effects of mannitol on diuresis and acid–base equilibrium in critically ill mechanically ventilated patients, according to the Stewart approach. Plasma and urine electrolyte concentrations were analysed using a novel point-of-care medical device capable of performing a semiquantitative urine analysis.

## Material and methods

### Study population

This prospective observational study was conducted in the general ICU of the ASST Santi Paolo Carlo, San Paolo University Hospital, Milan, Italy from October 2024 to February 2025. All consecutive sedated and mechanically ventilated patients requiring a mannitol infusion for clinical purposes as determined by the attending physician, were enrolled. Patients were excluded if they had chronic or acute renal failure, had previously received diuretics administration, were experiencing oliguria, hypovolemia, congestive heart failure, haemodynamic instability or cerebral haemorrage.

The study was approved by the institutional review board the Comitato Etico Territoriale Lombardia 1 (protocol number CET 237-2024) and informed consent was obtained in accordance with Italian regulations.

### Study protocol

Every patient received an infusion of 18% Mannitol at a rate of 1 g per kilogram of ideal body weight was administered over 20 min via a dedicated intravenous line. Throughout the study, the level of sedation and the mechanical ventilation settings (positive end-expiratory pressure—PEEP and minute ventilation) remained unchanged. Each patient received an infusion of 80 mL/kg of Ringer’s lactate solution. Neither the intravenous infusion nor the enteral nutrition was altered during the study.

### Data collection

The anthropometric, demographic and clinical characteristics of the patients were recorded upon enrolment.

Before mannitol infusion (T_0_), plasma acid–base variables, total diuresis and hemodynamic data were obtained. At the end of the mannitol infusion (T_END_) and then every 60 min (T_60_, T_120_ and T_180_), plasma acid–base variables and hemodynamics were obtained. Urinary electrolytes were collected at the end of the mannitol infusion (T_END_) and after every 30 min (T_30_, T_60_, T_90_, T_120_, T_150_ and T_180_). Hourly diuresis was obtained before mannitol infusion and total diuresis was measured from the end of the infusion (T_END_) until the end of the study. Urinary urea nitrogen (UUN) was obtained hourly from the end of the mannitol infusion.

Plasma acid–base variables (arterial pH; arterial carbon dioxide partial pressure—PaCO_2_, standard base excess—SBE), electrolytes (plasma sodium concentration—p[Na^+^], plasma potassium concentration—p[K^+^], plasma calcium concentration—p[Ca^++^], plasma chloride concentration—p[Cl^−^]) and lactate concentration (p[Lac^−^]) were obtained via an arterial blood sample and subsequent blood gas analysis using the RapidPoint® 500 system (Siemens Healthineers, Erlangen, Germany). Plasma albumin, creatinine and blood urea nitrogen (BUN) concentrations were also determined using standard laboratory methods.

Urine variables (urine pH; urine sodium concentration—u[Na^+^], urine potassium concentration—u[K^+^], urine ammonium concentration—u[NH_4_^+^], urine chloride concentration—u[Cl^−^]) and total diuresis were obtained from a spot urine sample via a urinary catheter and subsequent analysis by the KING monitoring system (King Instant MonitorinG Kures, Milan, Italy). The KING system provides a real-time monitoring of key renal parameters (urine Na^+^, K^+^, Cl^–^, NH4^+^, and pH). It was connected to the patient’s urinary catheter and the analyzer’s measuring principle was based on the potentiometric method using ion-sensitive sensors. The analyses were obtained without any dilution process.

Urinary urea nitrogen (UUN) was also determined using standard laboratory methods.

### Derived variables

Plasma strong ion difference (pSID) was computed as [13, 14]:

$$\text{pSID}=\text{p}\left[{\text{Na}}^{+}\right]+\text{p}\left[{\text{K}}^{+}\right]-\text{p}\left[{\text{Cl}}^{-}\right]-\text{p}\left[{\text{Lac}}^{-}\right]$$

Urine strong ion difference (uSID) was computed as [13]:

$$\text{uSID}=\text{u}\left[{\text{Na}}^{+}\right]+\text{u}\left[{\text{K}}^{+}\right]+\text{u}\left[{\text{NH}}_{4}^{+}\right]-\text{u}\left[{\text{Cl}}^{-}\right]$$

Urine osmolarity (uOsm) was calculated as [15]:

$${\text{uOsm}} = 2 \times ({\text{u}}[{\text{Na}}^{ + } ] + {\text{u}}[{\text{K}}^{ + } ]) + {\raise0.7ex\hbox{${{\text{UUN}}}$} \!\mathord{\left/ {\vphantom {{{\text{UUN}}} {2.8}}}\right.\kern-0pt} \!\lower0.7ex\hbox{${2.8}$}}$$

where UUN is urinary urea nitrogen in mg/dL.

Plasma osmolality (pOsm) was calculated as [15]:

$${\text{pOsm}} = 2 \times {\text{u}}[{\text{Na}}^{ + } ] + {\raise0.7ex\hbox{${{\text{Glu}}}$} \!\mathord{\left/ {\vphantom {{{\text{Glu}}} {18}}}\right.\kern-0pt} \!\lower0.7ex\hbox{${18}$}} + {\raise0.7ex\hbox{${{\text{BUN}}}$} \!\mathord{\left/ {\vphantom {{{\text{BUN}}} {2.8}}}\right.\kern-0pt} \!\lower0.7ex\hbox{${2.8}$}}$$

where Glu is plasma glucose concentration in mg/dL and BUN in blood urea nitrogen in mg/dL.

Free water clearance (C_H2O_) was computed as [16]:

$${\text{C}}_{{{\text{H}}_{2} {\text{O}}}} = {\text{UFR}} - ({\raise0.7ex\hbox{${{\text{uOsm}}}$} \!\mathord{\left/ {\vphantom {{{\text{uOsm}}} {{\text{pOsm}}}}}\right.\kern-0pt} \!\lower0.7ex\hbox{${{\text{pOsm}}}$}} \times {\text{UFR}})$$

where UFR is urine flow rate.

### Statistical analysis

Continuous data are reported as mean ± standard deviation or median [interquartile range], as appropriate; categorical data are reported as number (percentage). One-way ANOVA for repeated measures or Friedman Test were used to investigate the difference within measurement timepoints in terms of plasma and urine variables and hemodynamic data; a *post-hoc* pairwise comparison with Bonferroni correction was subsequently applied, when appropriate. A two-ways ANOVA was used to compare the response to mannitol infusion (*p*_*INT*_) between patients with higher or lower urine output (*between effect*, based on the median value of urinary output at T_0_—*p*_*UO*_) along the measurement timepoints (*within effect* – *p*_*TIME*_), using patients as *random effects*.

Statistical analysis and figures were performed using R Studio (RStudio. Integrated Development for R. RStudio, PBC, Boston, USA).

## Results

A total of 42 consecutive patients were enrolled and their baseline characteristics are presented in Table 1. None of the patients received vasopressors or inotropes. The mean infusion dosage of mannitol was 62 ± 4 g and patients were ventilated with a mean minute ventilation of 7.1 ± 1.6 L/min and with a median PEEP level of 5 [5–8] cmH_2_O throughout the study. Accordingly, PaCO_2_ did not change throughout the study.

**Table 1:** Baseline characteristics of the study population

	*N* = 42
Age, years	54 ± 19
Male sex, % (n)	67 (28)
Body mass index, kg/m^2^	27 ± 6
SAPS II score	31 [24–40]
APACHE III score	10 [7–13]
Albumin, g/dL	2.7 ± 0.2
Creatinine, mg/dL	0.6 [0.5–0.8]
Glomerular filtration rate, mL/min	136 ± 58
Admission diagnosis, % (n)	
Acute cardiogenic pulmonary edema	12.0 (5)
Hypoxemic respiratory failure	40.5 (17)
Sepsis	21.4 (9)
Post-surgical	7.1 (3)
Other	19.0 (8)
FiO_2_	45 ± 15
PEEP, cmH_2_O	5 [5–8]
Tidal volume, mL	500 ± 70
Respiratory rate, bpm	14 [12–15]
Minute ventilation, L/min	7.1 ± 1.6
Airway peak pressure, cmH_2_O	22 [20–26]
Airway plateau pressure, cmH_2_O	17 [15–19]
Mean airway pressure, cmH_2_O	10 [8–12]
PaCO_2,_ mmHg	42 ± 7
PaO_2,_ mmHg	83 [71–98]
PaO_2_/FiO_2_	190 [144–263]

*SAPS* simplified acute physiology score, *APACHE* acute physiologic assessment and chronic health evaluation, *FiO*_*2*_ inspired oxygen fraction, *PEEP* positive end-expiratory pressure, *PaCO*_*2*_ arterial carbon dioxide partial pressure, *PaO*_*2*_ arterial oxygen partial pressure

### Whole population: diuresis and plasma

**Fig. 1:**
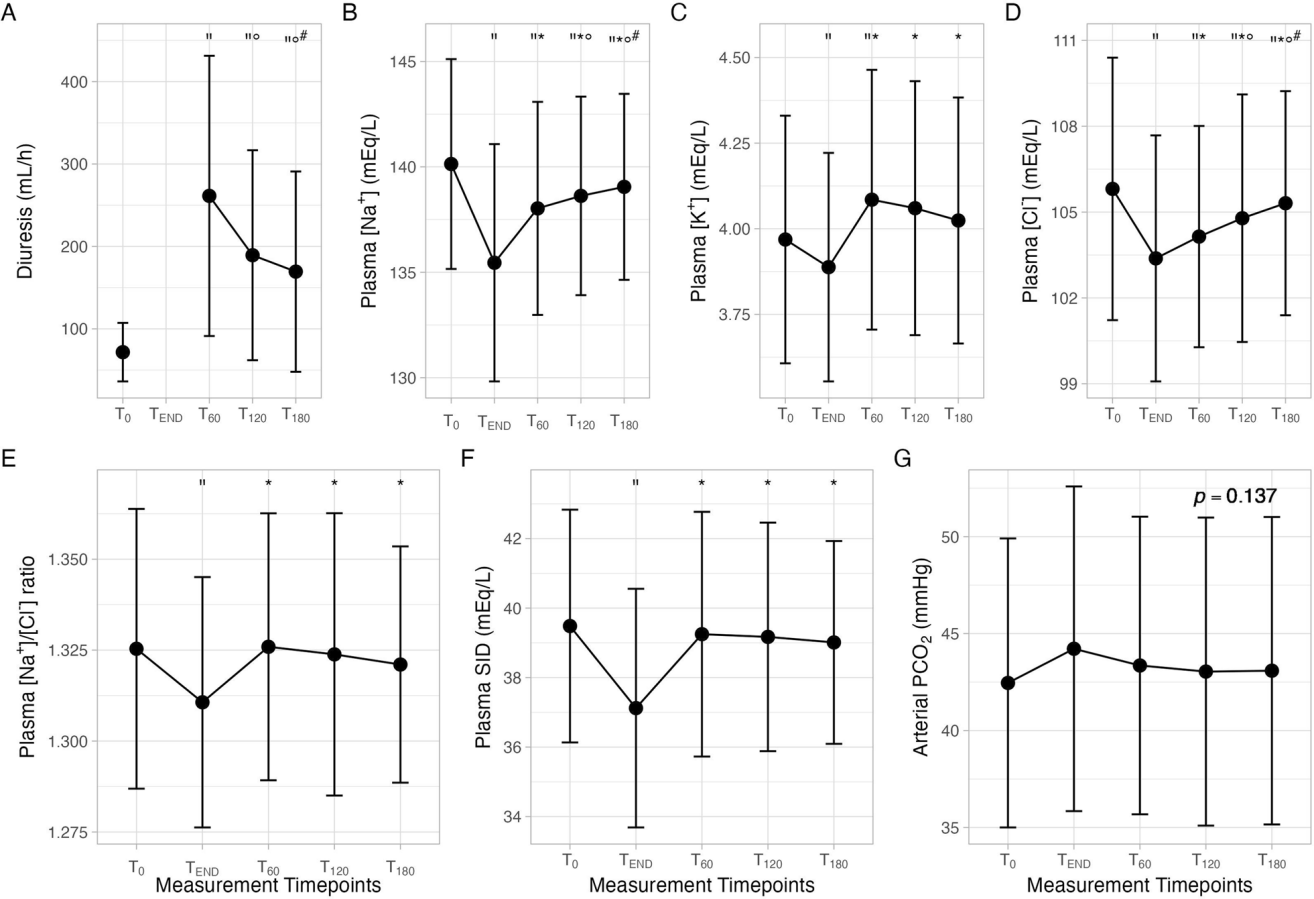
Time course of hourly diuresis (**A**), plasma electrolytes (sodium—**B**, potassium—**C** and chloride—**D**), plasma strong ion difference (**E**) and arterial carbon dioxide partial pressure (**F**) before mannitol infusion (T_0_), at the end of mannitol infusion (T_END_) and after 60 (T_60_), 120 (T_120_) and 180 (T_180_) minutes from the end of mannitol infusion. “: *p* < 0.05 vs T_0_; *: *p* < 0.05 vs T_END_; °: *p* < 0.05 vs T_60_; ^#^: *p* < 0.05 *vs* T_120_

Diuresis at the end of the mannitol infusion (T_END_) was 150 mL/h, then significantly decreased for up to 180 min, remaining higher compared to baseline (T_0_) (Table 2; Fig. 1).

**Table 2:** Time-course of plasma acid–base variables and electrolytes

*N* = 42	T_0_	T_END_	T_60_	T_120_	T_180_	*p*
Arterial pH	7.43 ± 0.05	7.40 ± 0.06”	7.41 ± 0.05*	7.42 ± 0.06*	7.42 ± 0.06*°	** < 0.001**
PaCO_2_, mmHg	42 ± 6	44 ± 8	43 ± 8	43 ± 8	43 ± 8	0.137
PaO_2_, mmHg	85 ± 20	83 ± 17	80 ± 15	84 ± 19	83 ± 21	0.116
PaO_2_/FiO_2_	182 ± 65	177 ± 54	167 ± 51	175 ± 54	171 ± 62	0.239
[HCO_3_^−^], mMol/L	26 ± 2.7	27.5 ± 2.8	27.8 ± 2.9	27.9 ± 3.1	28.4 ± 3.1	0.407
Base Excess, mMol/L	2.3 ± 3.0	1.7 ± 2.8”	2.1 ± 2.7*	2.3 ± 2.8*	2.4 ± 2.9″*°^#^	** < 0.001**
p[Na^+^], mEq/L	139 ± 4	135 ± 4”	137 ± 4″*	138 ± 4″*°	138 ± 3″*°^#^	** < 0.001**
p[K^+^], mEq/L	3.91 ± 0.2	3.8 ± 0.2”	4.0 ± 0.3*”	4.0 ± 0.3*	4.0 ± 0.3*	** < 0.001**
p[Ca^++^], mEq/L	1.14 ± 0.04	1.10 ± 0.05”	1.13 ± 0.05″*	1.14 ± 0.05*°	1.14 ± 0.05*°^#^	** < 0.001**
p[Cl^−^], mEq/L	105 [101–107]	103 [101–105]”	104 [101–106]”*	104 [102–107]”*°	104 [102–107]”*°^#^	** < 0.001**
pSID, mEq/L	39.5 ± 3.3	36.0 ± 3.4”	38.0 ± 3.6*	37.9 ± 3.3*	37.9 ± 2.9*	** < 0.001**
Albumin, g/L	2.6 ± 2	2.6 ± 2	2.7 ± 2	2.7 ± 3	2.7 ± 2	0.380
Hemoglobin, g/dL	10.5 ± 1.6	9.7 ± 1.6”	10.5 ± 1.4*	10.6 ± 1.4*^#^	10.7 ± 1.3*°^#^	** < 0.001**
Lactates, mMol/L	1.1 ± 0.5	0.9 ± 0.2	0.9 ± 0.2	0.9 ± 0.2	0.9 ± 0.2	0.366
Glucose, mg/dL	124 ± 22	122 ± 25	124 ± 27	126 ± 32	127 ± 29	0.229
FWC, mL/min	− 2.3 ± 3.1	–	0.4 ± 1.3”	− 0.1 ± 0.9″°	− 0.5 ± 1.4″°^#^	** < *0.001***

*PaCO*_*2*_ arterial carbon dioxide partial pressure, *PaO*_*2*_ arterial oxygen partial pressure, *FiO*_*2*_ inspired oxygen fraction, *[HCO*_*3*_^*−*^*]* plasma bicarbonate concentration, *p[Na*^*+*^*]* plasma sodium concentration, *p[K*^*+*^*]* plasma potassium concentration, *p[Ca*^*++*^*]* plasma calcium concentration, *p[Cl*^*−*^*]* plasma chloride concentration, *pSID* plasma strong ion difference

”: *p* < 0.05 vs T_0_; *: *p* < 0.05 vs T_END_; °: *p* < 0.05 vs T_60_; ^#^: *p* < 0.05 vs T_120_. Bold: significant *p*

Hemoglobin, plasma sodium and chloride concentrations decreased significantly after the infusion ended (T_END_), compared to T_0_: comparing T_0_ to T_END_, hemoglobin decreased from 10.5 ± 1.6 to 9.7 ± 1.6 g/dL, plasma sodium decreased from 139 ± 4 to 135 ± 4 mEq/L and plasma chloride decreased from 105 [103–108] to 103 [101–105] mEq/L. At subsequent time points, hemoglobin, plasma sodium and chloride concentrations significantly increased compared to T_END_; notably, plasma sodium and chloride concentrations remained lower than baseline levels (T_0_) (Table 2; Fig. 1).

According to the Stewart approach, arterial pH decreased significantly at the end of the mannitol infusion (T_END_) as compared to T_0_ (from 7.43 ± 0.05 to 7.40 ± 0.06) due to a reduction in plasma SID (from 39.5 ± 3.3 at T_END_ to 36.0 ± 3.4 mEq/L at T_0_). After 60–120-180 min from the end of the infusion (T_60_ T_120_ T_180_), arterial pH and plasma SID were higher compared to T_END_ and returned to their baseline values.

### Whole population: urine

**Fig. 2:**
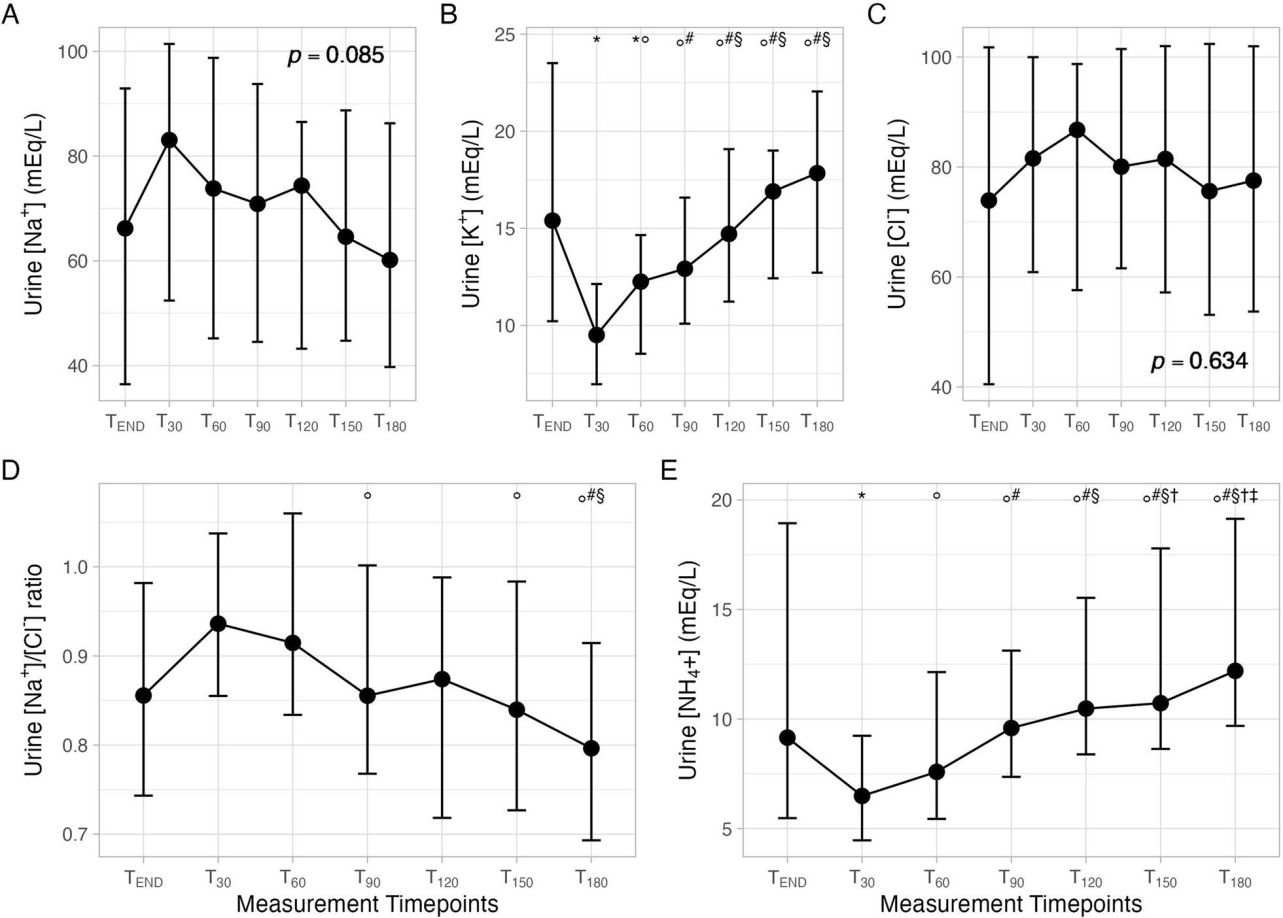
Time course of hourly diuresis (**A**), plasma electrolytes (sodium—**B**, potassium—**C** and chloride—**D**), plasma strong ion difference (**E**) and arterial carbon dioxide partial pressure (**F**) before mannitol infusion (T_0_), at the end of mannitol infusion (T_END_) and after 60 (T_60_), 120 (T_120_) and 180 (T_180_) minutes from the end of mannitol infusion. “: *p* < 0.05 vs T_0_; *: *p* < 0.05 vs T_END_; °: *p* < 0.05 vs T_60_; ^#^: *p* < 0.05 *vs* T_120_

Urinary sodium and chloride concentrations remained unchanged throughout the study (see Table 3; Fig. 2). A T_30_ urinary SID decreased due to a decreased urinary ammonium concentration compared to the end of the infusion (T_END_), then both urinary SID and urine ammonium concentration increased significantly since 60 min after the end of mannitol infusion (T_60_) and remained significantly higher for a period of up to 180 min (T_180_) compared to T_30_.

**Table 3:** Time-course of urinary acid–base variables and electrolytes

*N* = 42	T_END_	T_30_	T_60_	T_90_	T_120_	T_150_	T_180_	*p*
Urinary pH	5.9 [5.5–6.6]	6.2 [5.4–6.7]	6.2 [5.3–6.8]	6.0 [5.2–6.75]	6.1 [5.3–6.8]	6.0 [5.2–6.7]	5.8 [5.3–6.8]	0.329
u[Na^+^], mEq/L	66 [36–93]	83 [50–102]	74 [45–99]	71 [44–95]	74 [42–87]	65 [44–89]	60 [38–87]	0.085
Absolute Na excretion, mEq	–	13 [4–20]	6 [3–17]°	6 [2–11]°^#^	5 [1–8]°^#^	3 [1–7]°^#§†^	3 [2–7]°^#§†^	** < 0.001**
u[K^+^], mEq/L	15 [10–24]	10 [7–12]*	12 [9–15]*°	13 [10–17]°^#^	15 [11–19]°^#§^	17 [12–19]°^#§^	18 [13–22]°^#§^	** < 0.001**
Absolute K excretion, mEq	–	2 [1, 2]	1 [1, 2]	1 [1, 2]	1 [1, 2]	1 [1, 2]	1 [1, 2]	0.052
u[Cl^−^], mEq/L	74 [40–102]	82 [61–101]	87 [56–99]	80 [61–102]	81 [57–102]	76 [53–103]	78 [53–103]	0.634
Absolute Cl excretion, mEq	–	14 [5–22]	7 [4–17]	8 [4–13]	6 [2–13]	4 [2–8]	4 [2–12]	** < 0.001**
u[NH_4_^−^], mEq/L	9 [5–19]	6 [4–9]*	8 [5–12]°	10 [7–13]°^#^	10 [8–16]°^#§^	11 [9–18]°^#§†^	12 [10–19]°^#§ †‡^	** < 0.001**
Absolute NH_4_ excretion, mEq	–	1 [0 – 2]	1 [1, 2]	1 [1–1]	1 [0 – 1]^#^	1 [0 – 1]	1 [1, 2]	** < 0.001**
uSID, mEq/L	21 ± 20	15 ± 10	16 ± 10	18 ± 10°	19 ± 12°^#§^	21 ± 11°^#§^	24 ± 13°^#§†‡^	** < 0.001**
Diuresis, mL/h	–	150 [96–231]	92 [67–189]°	90 [62–137]°	80 [50–126]°^#§^	64 [41–97]°^#§†^	68 [40–111]°^#§^	** < *0.001***

*u[Na*^*+*^*]* urinary sodium concentration, *u[K*^*+*^*]* urinary potassium concentration, *u[Ca*^*++*^*]* urinary calcium concentration, *u[Cl*^*−*^*]* urinary chloride concentration, *uSID* urinary strong ion difference, *FWC* free water clearance

*: *p* < 0.05 vs T_END_; °: *p* < 0.05 vs T_30_; ^#^: *p* < 0.05 vs T_90_; ^§^: *p* < 0.05 vs T_120_; ^†^: *p* < 0.05 vs T_120_; ^‡^: *p* < 0.05 vs T_120_. Bold: significant *p*

However, absolute ammonium excretion and the ratio between urinary Na and Cl concentration remained clinically unchanged throughout the study,

Free water clearance increased significantly 60 min after the end of the mannitol infusion (T_60_) compared to baseline (T_0_). It subsequently decreased, but never returned to baseline values by the end of the study (Table 2; Fig. 2).

### Patients with higher vs. lower urine output

Patients with higher (≥ 93 mL/h) or lower (< 93 mL/h) urine output presented a similar increase in arterial pH at T_180_ compared to T_0_, due to a similar increase in plasma SID. Additionally, base excess and hemoglobin concentration increased similarly after 180 min from baseline (T_0_).

Urinary SID and its determinants (urine sodium, potassium, ammonium and chloride) did not change after 180 min from the end of the infusion (Table 4). Free water clearance demonstrated similar behavior in two groups.

**Table 4:** Time-course of plasma and urine acid–base variables and electrolytes according to higher (≥ 93 mL/h) or lower urine output (< 93 mL/h)

*N* = 42	UO < 93 mL/h	UO ≥ 93 mL/h	*p*_UO_	*p*_TIME_	*p*_INT_
Arterial pH			0.127	** < 0.001**	0.769
T_0_	7.38 ± 0.06	7.41 ± 0.06			
T_180_	7.41 ± 0.05	7.44 ± 0.06			
PaCO_2_, mmHg			0.379	0.064	0.527
T_0_	45 ± 8	43 ± 9			
T_180_	44 ± 7	42 ± 8			
PaO_2_, mmHg			0.071	0.939	0.990
T_0_	88 ± 18	78 ± 15			
T_180_	88 ± 25	78 ± 17			
[HCO_3_^−^], mMol/L			0.898	0.051	0.874
T_0_	26.5 ± 2.3	26.4 ± 2.6			
T_180_	27.0 ± 2.9	27.0 ± 2.8			
Base Excess, mMol/L			0.326	** < 0.001**	0.996
T_0_	1.2 ± 2.8	2.1 ± 2.7			
T_180_	2.5 ± 3.1	3.3 ± 2.9			
p[Na^+^], mEq/L			0.278	** < 0.001**	0.249
T_0_	134 ± 4	135 ± 5			
T_180_	138 ± 3	139 ± 3			
p[K^+^], mEq/L			0.662	**0.001**	**0.025**
T_0_	3.9 ± 0.2	3.8 ± 0.2			
T_180_	4.0 ± 0.4	4.1 ± 0.2°			
p[Cl^−^], mEq/L			0.542	** < 0.001**	0.080
T_0_	103 ± 3	102 ± 3			
T_180_	105 ± 3	104 ± 3			
pSID, mEq/L			0.408	** < 0.001**	0.015
T_0_	37.3 ± 3.4	37.0 ± 3.6			
T_180_	38.1 ± 2.8	39.3 ± 2.8°			
Albumin, g/dL			0.159	**0.017**	0.191
T_0_	2.7 ± 0.3	2.8 ± 0.2			
T_180_	2.7 ± 0.3	2.9 ± 0.3			
Haemoglobin, g/dL			** < 0.001**	** < 0.001**	0.489
T_0_	8.9 ± 1.5	10.5 ± 1.2			
T_180_	10.0 ± 1.2	11.4 ± 1.1			
Lactates, mMol/L			0.554	0.670	0.933
T_0_	0.9 ± 0.2	1.0 ± 0.2			
T_180_	0.9 ± 0.2	1.0 ± 0.2			
Glucose, mg/dL			0.426	0.192	0.899
T_0_	120 ± 17	128 ± 34			
T_180_	125 ± 21	131 ± 35			
FWC, mL/min			0.876	0.543	0.987
T_0_	− 2.2. ± 3.0	− 2.4 ± 3.0			
T_180_	− 0.3 ± 1.0	− 0.6 ± 1.5			
uNa^+^], mEq/L			0.650	0.788	0.151
T_END_	64 ± 43	62 ± 24			
T_180_	56 ± 37	67 ± 33			
u[K^+^], mEq/L			0.818	0.173	0.108
T_END_	16 ± 8	13 ± 10			
T_180_	16 ± 5°	20 ± 9			
u[NH_4_^+^], mEq/L			0.209	0.326	0.163
T_END_	13.7 ± 8.7	9.2 ± 7.4			
T_180_	13.0 ± 6.4	12.8 ± 6.3			
u[Cl^−^], mEq/L			0.744	0.086	0.066
T_END_	69 ± 43	62 ± 23			
T_180_	68 ± 37	82 ± 31			
uSID, mEq/L			0.905	0.368	0.121
T_END_	73 ± 49	64 ± 34			
T_180_	56 ± 39	68 ± 25			
Diuresis, mL/h			** < 0.001**	** < 0.001**	** < 0.001**
T_0_	31 ± 22	71 ± 48*			
T_180_	24 ± 20	36 ± 41°			

*UO* urine output, *PaCO*_*2*_ arterial carbon dioxide partial pressure, *PaO*_*2*_ arterial oxygen partial pressure, *[HCO*_*3*_^*−*^*]* plasma bicarbonate concentration, *p[Na*^*+*^*]* plasma sodium concentration, *p[K*^*+*^*]* plasma potassium concentration, *p[Ca*^*++*^*]* plasma calcium concentration, *p[Cl*^*−*^*]* plasma chloride concentration, *pSID* plasma strong ion difference, *FWC* free water clearance, *u[Na*^*+*^*]* urinary sodium concentration, *u[K*^*+*^*]* urinary potassium concentration, *u[Ca*^*++*^*]* urinary calcium concentration, *u[Cl*^*−*^*]*: urinary chloride concentration, *uSID* urinary strong ion difference, *p*_UO_ *between* effect of urine output lower or higher than 93 mL/h, *p*_*TIME*_ *within* effect of time, *p*_*INT*_ interaction between urine output and time effects

*: *p* < 0.05 vs UO < 93 mL/h; °: *p* < 0.05 vs T_0_. Bold: significant *p*

## Discussion

The present study showed the plasma and urinary physiochemical effects of an intravenous administration of mannitol in mechanically ventilated patients: (1) diuresis increased significantly immediately after the end of the infusion, then decreased up to 180 min; (2) haemoglobin, plasma sodium and chloride concentrations decreased significantly after the end of the infusion, then significantly increased; (3) arterial pH as well as plasma SID decreased significantly at the end of the infusion resulting in dilutional acidosis; and (4) urinary SID and urinary ammonium concentration initially decreased, then after 60 min both increased significantly.

The most commonly used diuretics for critically ill patients are loop diuretics (*e.g.,* furosemide) and osmotic diuretics (e.g., mannitol). Despite their different clinical, pharmacological and pharmacokinetic characteristics, they significantly improve diuresis [17, 18].

In addition, both can significantly affect the composition of plasma and urine, leading to different alterations in acid–base equilibrium [11–14, 19]. According to the Stewart approach, an intravenous infusion of 40 mg of furosemide was found to significantly decrease the plasma chloride concentration and increase the plasma SID in critically ill patients. It was also found to increase urinary losses of all electrolytes and reduce urinary SID [20].

Mannitol was first used in 1945 to improve renal function in dogs following a period of ischaemia [21]. It is currently commonly administered in several clinical settings, including cases of drug intoxications, refractory oedema, oliguric renal failure and cerebral oedema. It is also used to prevent acute renal failure in patients with rhabdomyolysis or following the use of radiocontrast agents [2, 7, 17, 18, 22, 23]. Possible side effects include volume depletion due to its strong osmotic diuretic effect, as well as hypernatremia and metabolic acidosis, particularly with cumulative dosages and in cases of acute or chronic kidney failure [23–26]. Despite its common use in daily clinical practice, there is a paucity of studies that have described the physiological effects of mannitol in critically ill patients [17].

In the present study, the whole acid–base equilibrium of all patients was evaluated by simultaneously assessing plasma and urinary SID using an arterial blood gas analysis and analyzing urine composition by the K.IN.G analyzer [27].

The mannitol infusion immediately after 30 min provided a significant increase in diuresis compared to baseline, which remained significantly higher up to 180 min, yet significantly reduced compared to T_30_. A previous study reported that, for a similar dosage of mannitol administered to healthy subjects, urine flow increased from 78 ± 29 to 287 ± 21 mL/h at 90 min, with peak serum concentration occurring after 15 min [28].

At the end of mannitol infusion, plasma electrolyte concentrations decreased due to a dilutional effect, resulting in a reduction in SID. The same degree of dilution should be expected for Atot (mainly albumin and phosphate), with an alkalising influence that should partially compensate for the acidifying influence of the SID reduction. However, albumin levels remained constant, as well as arterial carbon dioxide levels, due to an unchanged ventilation throughout the study. Therefore, the predominant effect was the dilution of the SID, resulting in a dilutional metabolic acidosis [29–31].

Subsequently, at T_60_ the dilutional effect ended, in fact plasma electrolyte concentrations, pH and SID exhibited higher levels up to 180 min.

Similarly, the addition of mannitol to a priming solution for cardiopulmonary bypass resulted in an increase in diuresis and a decrease in serum sodium concentration (138 ± 28 to 133 ± 2.6 mEq/L) [32]. The duration of blood volume increase depends on the equilibrium rate of mannitol in the extracellular compartments and the renal excretion rate due to the osmotic diuresis.

At the urinary level the excretion of sodium and chloride did not change throughout the study, while ammonium concentration decreased significantly 30 min after the end of the infusion compared to T_END_, causing a decrease in urinary SID. Then, 90 min after the end of the infusion, both urine ammonium concentration and urinary SID increased significantly and remained higher up to 180 min compared to T_30_. This increase in the SID could be interpreted as a compensatory mechanism exerted by the kidneys to counterbalance metabolic dilutional acidosis, or it could simply reflect only an initial increase in urine flow, followed by a subsequent reduction [11]. In fact, urinary ammonium concentration depends on how much ammonium the kidney produces (in response to acidosis) and on how diluted or concentrated the urine is. In our study, even though ammonium concentration significantly decreased and then increased, the absolute amount of ammonium excreted remained clinically unchanged at the different timepoints.

Moreover, both the plasmatic Na⁺/Cl⁻ and urinary Na/Cl ratios remained constant, meaning that the dilutional effect in plasma was prevalent, the kidney relative excretion pattern was unchanged, and any differences in ammonium excretion were just due to urine dilution or concentration.

To evaluate the possible influence of intravascular volume on the response to mannitol administration, the population was divided into two groups based on higher or lower urine output. The effects of mannitol infusion were found to be similar in both groups. The increase in free water clearance was independent from the baseline urine flow rate.

## Limitations

The possible limitations of this study are: (1) the absence of laboratory measurements of serum osmolarity, which could have provided a clearer explanation for the reduction in serum electrolytes, primarily related to volume expansion, (2) the use of a reduced formula to surrogate plasma SID, not accounting for calcium and magnesium concentration variations, (3) the duration of the study, which lasted only 180 min, (4) the lack of baseline urinary electrolytes.

## Conclusions

The present study showed that, at the end of the infusion, mannitol induced a significant increase in diuresis, a reduction in plasma electrolytes, SID and pH leading to a dilutional acidosis, and at the same time a decrease in urinary ammonium and urinary SID. After 60 min from the end of infusion, arterial pH and plasma SID increased, while diuresis, urinary ammonium and urinary SID decreased. No differences were found in the absolute amount of ammonium excreted, nor in the urinary Na/Cl ratio throughout the study.

The urinary physicochemical analysis has provided a unique contribute to interpret these changes as secondary to urine dilution or concentration, because the kidney’s relative excretion pattern has remained unchanged.

## Data Availability

The dataset analysed during the current study is available from the corresponding author on reasonable request.

## Abbreviations

RASS: Renin-angiotensin aldosterone system
SID: Strong ion difference
A_TOT_: Total weak acid concentration
PEEP: Positive end-expiratory pressure
PaCO_2_: Arterial carbon dioxide partial pressure
SBE: Standard base excess
p[Na^+^]: Plasma sodium concentration
p[K^+^]: Plasma potassium concentration
p[Cl^−^]: Plasma chloride concentration
p[Lac^.^]: Plasma lactate concentration
BUN: Blood urea nitrogen
u[Na^+^]: Urine sodium concentration
u[K^+^]: Urine potassium concentration
u[Cl^−^]: Urine chloride concentration
u[NH_4_^+^]: Urine ammonium concentration
UUN: Urinary urea nitrogen
uSID: Urinary strong ion difference
uOsm: Urine osmolality
pOsm: Plasma osmolality
C_H2O_: Free water clearance
